# Insights into the abundance and diversity of abyssal megafauna in a polymetallic-nodule region in the eastern Clarion-Clipperton Zone

**DOI:** 10.1038/srep30492

**Published:** 2016-07-29

**Authors:** Diva J. Amon, Amanda F. Ziegler, Thomas G. Dahlgren, Adrian G. Glover, Aurélie Goineau, Andrew J. Gooday, Helena Wiklund, Craig R. Smith

**Affiliations:** 1 Department of Oceanography, University of Hawai’i at Mānoa, 1000 Pope Road, Honolulu, HI 96822 USA; 2 Uni Research, Thormøhlensgate 55, 5008 Bergen, Norway; 3 Department of Marine Sciences, University of Gothenburg, Box 463, 40530 Gothenburg, Sweden; 4 Life Sciences Department, Natural History Museum, Cromwell Rd, London SW7 5BD, UK; 5 National Oceanography Centre, University of Southampton, Waterfront Campus, European Way, Southampton SO14 3ZH, UK

## Abstract

There is growing interest in mining polymetallic nodules in the abyssal Clarion-Clipperton Zone (CCZ) in the Pacific. Nonetheless, benthic communities in this region remain poorly known. The ABYSSLINE Project is conducting benthic biological baseline surveys for the UK Seabed Resources Ltd. exploration contract area (UK-1) in the CCZ. Using a Remotely Operated Vehicle, we surveyed megafauna at four sites within a 900 km^2^ stratum in the UK-1 contract area, and at a site ~250 km east of the UK-1 area, allowing us to make the first estimates of abundance and diversity. We distinguished 170 morphotypes within the UK-1 contract area but species-richness estimators suggest this could be as high as 229. Megafaunal abundance averaged 1.48 ind. m^−2^. Seven of 12 collected metazoan species were new to science, and four belonged to new genera. Approximately half of the morphotypes occurred only on polymetallic nodules. There were weak, but statistically significant, positive correlations between megafaunal and nodule abundance. Eastern-CCZ megafaunal diversity is high relative to two abyssal datasets from other regions, however comparisons with CCZ and DISCOL datasets are problematic given the lack of standardised methods and taxonomy. We postulate that CCZ megafaunal diversity is driven in part by habitat heterogeneity.

A combination of biological, chemical and geological processes has led to the formation of high abundances of polymetallic “manganese” nodules on the abyssal seafloor in the Clarion-Clipperton Zone (CCZ) of the Pacific Ocean^1^. These nodules are potentially valuable sources of cobalt, copper, manganese and nickel (among other metals), and this has led to an interest in mining this region^2^,^3^. The CCZ, a ~6 million km^2^ region bounded by the Clarion and Clipperton Fracture Zones, lies in Areas Beyond National Jurisdiction (ABNJ), and thus falls under the legal mandate of the International Seabed Authority (ISA)^4^. As of 2015, 16 nodule-mining exploration contract areas had been approved by the ISA within the CCZ, each up to 75,000 km^2^ in area (https://www.isa.org.jm/). The ISA has recently adopted an environmental management plan for the CCZ^5^,^6^. In addition to this, the ISA has stipulated that prior to exploitation, a benthic biological baseline study must be undertaken for each exploration contract area^7^,^8^.

The UK-1 exploration contract area, licensed to UK Seabed Resources Ltd. (UKSRL), is the easternmost contract area within the CCZ and encompasses ~58,000 km^2^ of seafloor (Fig. 1). The ABYSSLINE (Abyssal Baseline) Project has been designed to undertake benthic biological baseline studies in accordance with ISA environmental guidelines within the UK-1 area. This project is led by scientists from the University of Hawai’i at Mānoa (USA), and includes scientists from Hawai’i Pacific University (USA), the Natural History Museum, London (UK), the National Oceanography Centre, Southampton (UK), Senckenberg Gesellschaft für Naturforschung (Germany), Uni Research (Norway), and the International Research Institute of Stavanger (Norway). Specific aims include (1) evaluating baseline conditions of community structure and biodiversity for megafauna, macrofauna, meiofauna and microbes, (2) establishing how community structure, biodiversity and benthic carbon cycling vary across the UK-1 contract area and with environmental parameters such as nodule cover, and (3) assessing faunal population connectivity within the UK-1 contract area and across the CCZ.

The megafauna constitute an important component of the biodiversity in the abyssal deep sea and play a significant role in deep-sea ecosystem function (e.g., phytodetritus consumption and bioturbation)^9^. It is expected that nodule-mining operations will impact the megafauna within directly mined areas and over broader scales^3^,^10^,^11^,^12^. Physical removal of nodules, and their burial by sediment plumes, will remove hard-substrate habitats, destroying the nodule-obligate fauna without possible re-establishment over ecological time scales^3^,^10^,^12^,^13^,^14^. In addition, swathes of sediment >5 m width and 5–20 cm depth may be removed, compacted or re-suspended by machinery, possibly resulting in large footprints that would be further impacted by sediment plumes deposited on the benthos across wider areas^3^,^10^,^11^,^13^,^15^,^16^,^17^. Other likely consequences of deep-sea mining may include changes in the geochemistry of the sediment^18^, and light and noise pollution from machinery^13^. The cumulative effects of these operations have been hypothesised to be long-lasting, e.g., at least decades for sediment geochemistry and sediment community recovery^15^,^18^, and millennia for nodule regrowth and recovery of the nodule-obligate fauna^3^,^14^.

Biological research in the CCZ, and other abyssal Pacific nodule areas, was carried out in response to an initial upsurge of interest in mining in the late 1970s. Studies, including experimental disturbances, have been undertaken by contractors in several contract areas^14^,^19^,^20^,^21^,^22^,^23^,^24^,^25^,^26^,^27^. However, baseline patterns of megafaunal abundance, diversity and community structure remained poorly characterized across the CCZ. In the last ten years, there has been renewed interest in the area, with eight exploration contract areas being licensed by the ISA during this period. Despite increased prospecting and great advances in deep-sea sampling technology (e.g. high-definition cameras, Autonomous Underwater Vehicles) and analytical methods (e.g. DNA-based taxonomy), knowledge of the regional fauna across all size classes has not increased greatly^12^,^15^,^23^,^28^,^29^,^30^,^31^,^32^,^33^,^34^. No megafaunal studies had been undertaken in the UK-1 contract area prior to licensing in 2013. An Ocean Biogeographic Information System (OBIS) search for abyssal benthic megafaunal taxa inhabiting the UK-1 contract area revealed zero records^35^. When the OBIS search was widened to the entire eastern CCZ, an area of more than 1 million km^2^, it yielded only 34 records of megafaunal taxa^35^, highlighting the lack of available data from this region.

In order to predict and manage the environmental impacts of mining in the CCZ and within the UK-1 contract area, baseline knowledge of the abundance, diversity, and species ranges of megafauna is essential. Understanding spatial variations in megafaunal community structure, and their correlations with environmental parameters such as nodule cover, density and size, is also crucial. Here, we present the first baseline data for megafauna in the UK-1 exploration contract area based on ROV surveys and samples collected during the first cruise of the ABYSSLINE Project in 2013.

## Results

### Collected Megafauna

We collected 18 metazoan megafaunal individuals corresponding to 12 morphotypes and representing three metazoan phyla: Cnidaria (five individuals from five species), Echinodermata (11 individuals from five species) and Porifera (two individuals from two species) (Fig. 2 and see Supplementary Table S1). Preliminary results indicate that seven of the 12 species were new to science, including three new genera: *Abyssoprimnoa gemina* Cairns, 2015 (a new genus and species)^36^, two undescribed genera of the coral family Isididae, a new species of *Phelliactis* anemone, a new species of primnoid *Calyptrophora persephone* Cairns, 2015^36^, and two new species of *Caulophacus* sponges (Fig. 2 and see Supplementary Table S1). In addition to the morphological descriptions, DNA sequences from multiple markers (18S rRNA, 16S rRNA, mitochondrial CO1) were independently obtained from the megafaunal morphotypes, confirming through phylogenetic reconstruction that these are separate species^37^ (Dahlgren *et al.* 2016)^38^. In addition to the metazoans, a total of 28 xenophyophores (large monothalamous foraminifera of uncertain rank i.e., protistan megafauna), representing 14 putative species, was collected (Gooday, Holzmann & Goineau, in prep.). Nine of the species had a plate-like morphology and included only one described species, *Psammina limbata* Kamenskaya, Gooday Tendal & Melnik, 2015 (Fig. 2). The remaining five species had tests with lumpy or tubular morphologies, or formed more complex, three-dimensional lattice-like structures. None had reticulated tests.

### Image analysis

#### Morphotype richness

A total of 180 megafaunal morphotypes (metazoan and protistan) was identified in ROV video surveys during the AB01 cruise (both UK-1 Stratum A and the EPIRB sites) (Figs 2,3 and Supplementary Table S2) (Amon *et al.*, in prep.). This is likely an underestimate due to poor image resolution, difficulty identifying fauna from images, and the presence of cryptic species. There were 170 morphotypes observed within the UK-1 contract area and 132 seen at the EPIRB site, with 122 observed at both locations. Forty-eight morphotypes were only observed within the UK-1 contract area, whereas ten were noted only at the EPIRB site (see Supplementary Table S2). This difference in morphotype richness is likely due to a higher sampling effort in UK-1 rather than from distinct species ranges. Cnidaria was the most species-rich phylum with a total of 48 morphotypes observed during AB01, followed by the Echinodermata (41 morphotypes) (Fig. 3). Although 180 morphotypes have been recognized, species-accumulation curves suggest that megafaunal morphotype richness has not been fully characterized in areas surveyed during AB01 (Fig. 4). Chao-1 estimated 196 (s.d. = 9) total species, whereas Bootstrap and Jacknife2 estimated 198 and 236 morphotypes respectively. For UK-1 Stratum A alone, Chao-1 estimated that there are 208 (s.d. = 12.7) species, whereas Bootstrap and Jacknife2 estimated 192 and 229 morphotypes, respectively.

Morphotypes observed during AB01 were also assigned to functional groups based on mobility (sessile or mobile) and substrate utilization (obligate hard-substrate, obligate soft-sediment, facultative i.e., seen on both hard and soft substrates, or bentho-pelagic fauna). Sessile morphotypes constituted 63.5% of morphotype richness, while 36.5% were mobile. Obligate hard-substrate dwellers constituted 51.9% of morphotype richness, facultative species constituted 36.5%, obligate soft-sediment dwellers 6.1%, and bentho-pelagic morphotypes 5.5%. Chao-1 richness estimates by substrate utilization showed that obligate hard-substrate fauna contributed approximately 50% of all morphotypes observed (Fig. 4).

#### Patterns of megafaunal abundance, community structure, and diversity

We counted 6241 individuals from 136 morphotypes during four quantitative transects (total area = 4204 m^2^) yielding a mean megafaunal abundance of 1.48 ind. m^−2^ (s.e. = 0.03) or 14,000 ind. ha^−1^ (Table 1). Although these transects were at both EPIRB site and Site 6, this analysis had 44 fewer morphotypes than were observed in the qualitative analysis which used all available imagery from the AB01 cruise. At the scale of individual frames (i.e., using frames as replicates), mean abundance varied significantly between transects within site EPIRB (H = 5.125, p = 0.024) and between transects at Site 6 and EPIRB (p < 0.0001) but not between transects within Site 6 (H = 0.012, p = 0.915). Mean abundance did not vary significantly when data were pooled by transect or site. The xenophyophores were the most abundant group (0.65 ind. m^−2^) with nearly double the abundance of Cnidaria, the second-most abundant group (Fig. 3). Ninety-two percent of Cnidaria abundance consisted of alcyonaceans from nine morphotypes.

The five most abundant morphotypes across all quantitative transects were the ophiuroid, *Ophiomusium* cf. *glabrum*, three plate-like xenophyophore morphotypes, and the primnoid *Abyssoprimnoa gemina* (Table 2 and Fig. 2). The dominant species varied to some degree between transects, with *Ophiomusium* cf. *glabrum* and xenophyophore plate-like morphotype 2 occurring among the top five species across all transects (Table 2). The five most abundant morphotypes observed during AB01 dominated the megafaunal community, comprising nearly half (45%) of all individuals. The remaining morphotypes were generally rare, as indicated by the rank order abundance curve (see Supplementary Figure S1), with 62% of the morphotypes (84 of 136) observed fewer than ten times, and 24% (33 morphotypes) seen only once in quantitative transects. There was greater community similarity at the transect level within sites (>80% similarity) than between sites (<68% similarity) (see Supplementary Figure S2). There were no significant differences between transects or sites for a range of diversity indices (Table 1).

#### Abundance and diversity of nodules and nodule fauna

A total of 23,467 nodules was counted in 241 frames from the UK-1 and EPIRB sites. The exposed plan area of individual nodules ranged from 0.3-1041.4 cm^2^ (see Supplementary Table S3). Nodule density in individual frames ranged across two orders of magnitude, from 3 to 535 nodules m^−2^, and nodule cover (percent of seafloor plan area occupied by exposed nodules) ranged from 0.2 to 50%, i.e. from low to medium cover by CCZ standards (see Supplementary Table S3). Average surface area per nodule was significantly negatively correlated with nodule density (r = −0.324, p < 0.0001) and average nodule density was strongly positively correlated with nodule cover (%) (r = 0.850, p < 0.0001). At the individual frame scale, all nodule parameters were significantly different between sites (p < 0.005). Mean nodule area was significantly different between transects (p < 0.02), except between Transect 2 at Site 6 and Transect 1 at Site EPIRB. Mean nodule density per frame was significantly different (p < 0.01) between transects at Site 6 and Transect 2 at Site EPIRB. For nodule cover (%) by frame, only Transect 1 at Site EPIRB was significantly different from transects at Site 6 (p < 0.04). Variations were observed in the average sizes of nodules and their abundances by transect and by site (see Supplementary Table S3). At the transect level, there were no significant differences in average nodule size or percent nodule cover. However, nodule abundances differed significantly between transects at Site 6 (p < 0.01). At the site level, only nodule abundance was significantly different (χ2 = 22.286, p < 0.001), with the EPIRB site containing about twice as many nodules as the UK-1 site. It should be noted that nodule densities and nodule size apply only to nodules with exposed surfaces and are hence a minimum estimate; box cores collected in UK-1 Stratum A revealed that at any location, many of the nodules in the top 10 cm were partially or fully buried (Smith *et al.*, in prep.).

In the quantitative nodule surveys conducted during AB01, 148 megafaunal individuals were counted. The top five morphotypes, accounting for more than 50% of the total abundance, represented two major groups of sessile megafauna: plate-like xenophyophores and alcyonaceans. Hard-substrate-obligate megafauna observed throughout AB01 were mainly suspension feeders (89.4% of morphotypes) and were observed on nodules ranging in size from 2.4 to 1041.4 cm^2^ in exposed plan area. Megafaunal abundance at the frame level was weakly, but significantly, positively correlated with nodule abundance (r = 0.135, p = 0.036). Areas of low (<15%) nodule cover accumulated more species and had higher estimated total species richness (Chao 1, Bootstrap, Jacknife2) than areas of medium (15–50%) nodule cover (Fig. 5). There were no marked differences between the proportions of obligate soft-sediment dwellers (2.5% vs. 3.1%), facultative (66.3% vs. 59.1%) and obligate hard-substrate dwellers (31.3% vs. 37.8%) in areas of low versus medium nodule cover.

## Discussion

### Biodiversity of megafauna in the eastern CCZ

We provide the first insights into megafaunal abundance and diversity from the UK-1 exploration contract area and the eastern CCZ. Our results indicate that the megafauna of the UK-1 area and eastern CCZ is poorly characterized; seven of the 12 collected specimens were new to science, including the fourth most abundant organism observed during this study, the alcyonacean *Abyssoprimnoa gemina*. This faunal novelty occurs at high taxonomic levels, i.e., 25% of collected species belong to undescribed genera. Conversely, all of the previously-known species collected during AB01 occur widely throughout the CCZ, and in some cases, across multiple ocean basins, although molecular analyses may yet reveal the presence of cryptic species (see Supplementary Table S1). This is echoed in the morphotypes observed in the image analysis, with more than half (57%) appearing in other contract areas in the CCZ^20^,^21^,^22^ (http://ccfzatlas.com/).

The richness and abundance of megafauna within our surveys in the 30 × 30 km UK-1 Stratum A appear to be high (170 observed morphotypes and 1.31 ind. m^−2^) relative to other abyssal areas. However, there seem to be few comparable CCZ data as it is not clear whether similar criteria and standards were used for megafaunal morphotypes and also data collection (see discussion below). Species richness in this area remains undersampled indicating that more baseline data are required to better understand how these diverse megafaunal assemblages will be impacted by mining. Tilot^23^ appears to be the only study in the CCZ that included xenophyophores in species-richness estimates, resulting in 159 morphotypes from three sites in the central CCZ, ranging across a much greater area than ours, i.e., >10,000 km^2^ (~350 km of transects and >60,000 images). Removing protists (xenophyophores) from our species-richness estimates for UK-1 Stratum A to allow comparisons with other datasets reduces the number of morphotypes to 153. This is substantially more than the ~38 metazoan morphotypes observed in the COMRA contract area (10 hrs of video and 2000 images)^21^ and the ~70 metazoan morphotypes at the DOMES sites (~70,000 images)^22^ all in the central to western CCZ. The abundance of metazoan megafauna in the UK-1 Stratum A (0.83 ind. m^−2^) substantially exceeded megafaunal abundances observed in other contract areas in the CCZ: Chunsheng and Douding (2002) recorded mean metazoan megafaunal abundances of 0.01–0.02 ind. m^−2^ and Tilot (2006) recorded ~ 0.055 ind. m^−2^ from the NIXO 45 site. Vanreusel *et al.*^12^ surveyed the IFREMER, GSR, IOM and BGR exploration contract areas within the CCZ, and reported abundances of mobile and sessile fauna to be less than 0.5 ind. m^−2^, although they did not include xenophyphores, fish and crustacea, and likely underestimated fauna under 5 cm in size. Five morphotypes accounted for ~50% of the megafaunal individuals observed in AB01 data, while >25% of morphotypes were only observed once. A similar distribution of abundance among morphotypes was observed by Kamenskaya *et al.*^39^ in the Yuzhmorgeologiya contract area in the central CCZ. A long list of rare species is commonly observed across all size classes in the deep sea^40^ and likely results from undersampling of habitats combined with true rarity (e.g., very low population densities)^41^.

Xenophyophores were the most abundant group in our imaging surveys in the eastern CCZ during AB01 (~0.65 ind. m^−2^ or 44%) as also reported in the Yuzhmorgeologiya contract area, where they constitute 69% of the megafauna, albeit at much lower densities (0.16 ind. m^−2^)^31^,^39^. Thus, although xenophyophores have not been considered in most other megafaunal surveys in the CCZ, they appear to constitute a major fraction of megafaunal abundance across the region. A total of 28 specimens representing 14 species was recovered from boxcores and megacores during AB01 yielding an estimated density of ~5.2 ind. m^−2^ (Gooday and Goineau, unpublished data). However, some of the collected morphotypes were either too small to be seen in ROV images or would have been difficult to distinguish. These include an undescribed species of the genus *Aschemonella* with a dark grey ‘segmented’ tubular test (typically >2 cm in length) that is the most common xenophyophore in this part of the CCZ (Gooday and Goineau, unpublished observations). If only morphotypes resembling those visible in seafloor images are considered, then the estimated density reduces to 1.3 ind. m^−2^, which is closer to the estimate from the image analyses.

In a global context, megafaunal richness in our study areas are higher than two other, highly-productive, abyssal habitats worldwide. At Station M off California, 102 taxa were observed from an area three times larger than our study^42^ and 43 morphotypes were observed during a larger study (~6900 km^2^) in the Porcupine Abyssal Plain (PAP) that did not include xenophyophores^43^ (Table 1). Abundance estimates at the PAP were also an order of magnitude lower^43^ than the UK-1 area (Table 1). In contrast, abundances reported from Station M were higher than those reported here, and show a wide temporal variation^41^ (Table 1). Shannon’s Diversity (H’) and Pielou’s evenness (J’) in the UK-1 contract area were comparable to or higher than both Station M and the PAP in part because of the high-frequency of rare species^42^,^43^ (Table 1). Es(100–103) in the UK-1 contract area was roughly twice that at PAP^42^,^43^ (Table 1). Studies in the DISCOL area in the South Pacific recorded 170 morphotypes and abundance estimates of 0.041 ind. m^−2^, but this was from a much larger survey (over 700,000 m^2^) and also sampling methodologies may have differed substantially^14^,^19^,^27^. The species richness in our study areas was also comparable to a much larger area surveyed on the slopes of the Hawaiian archipelago spanning a depth range of 350–1500 m in the central Pacific Ocean (Vetter *et al.* 2010).

Megafaunal abundance, diversity and community composition were relatively homogenous between our transects and sites suggesting that abyssal megafaunal communities may be relatively homogenous on scales of one to hundreds of kilometers in the eastern CCZ. However, we hypothesize that substantial community heterogeneity exists at abyssal depths on the scale of individual nodules^26^,^30^, as well as over tens of meters as nodule cover varies, with bathymetric variation (e.g. on seamounts), and over regional scales (thousands of kilometers) as overlying productivity and vertical Particulate Organic Carbon (POC) flux change^9^,^44^.

### Megafaunal structuring factors in the eastern CCZ

Increased habitat heterogeneity has been shown to enhance species richness in many habitats^45^,^46^ and previous studies indicate that the presence of nodules influences the community composition, density and distribution of megafauna, macrofauna and meiofauna in the CCZ^23^,^25^,^30^,^32^. The presence of both abundant hard substrate (nodules) and soft substrate (sediments) contributes to high habitat heterogeneity in the CCZ, especially when compared to the regions of the deep sea lacking nodules or other hard substrate. This habitat heterogeneity should, in turn, enhance total species richness and diversity. The present study suggests that species richness and diversity in the UK-1 area are comparable to two more-productive abyssal regions, although any conclusions are somewhat tentative. Our results suggest that nodule cover does not need to be high to enhance species richness; once nodules were present, even in small numbers, they were colonised by numerous hard-substrate-obligate morphotypes. Thereafter, the increasing cover and volume occupied by nodules is hypothesized to reduce the habitat space for soft-sediment dwellers resulting in a decline in species richness (Fig. 5). This relationship is hypothesized to be unimodal. However, in the areas surveyed during AB01, nodule cover never exceeded 50%, potentially resulting in optimal proportions of both soft sediment and hard substrate (Fig. 5). Vanreusel *et al.*^12^ found that antipatharians and alcyonaceans were almost absent from nodule-free areas, indicating that the addition of hard substrate increased species richness and diversity.

Half of the morphotypes and 32% of the megafaunal individuals in UK-1 were hard-substrate obligates. Of the 26 xenophyophores recovered in box cores and megacores, 15 were attached to nodules and 11 occurred directly on the sediment, suggesting that nodules represent an important habitat for these protists as well^31^,^39^. Some of the collected species had low, encrusting morphologies and probably only live on hard substrates, but a few were also found on or in the sediment. Most of the xenophyophores in the UK-1 area are probably suspension feeders that colonize nodules opportunistically because these structures elevate them into the benthic boundary layer^47^. Similarly, most of the hard-substrate obligates (corals, bryozoans etc.) in this study appeared to be suspension feeders, as has been seen previously^25^,^29^,^30^,^48^. Thus, the presence of nodules served to increase trophic as well as taxonomic diversity. The feeding activities of these sessile suspension-feeding nodule dwellers may be particularly at risk from mining sediment plumes because they are adapted to benthic boundary layers with very low concentrations of suspended particles^47^ (Fig. 2). Furthermore, these sessile organisms are particularly at risk from mining activities as they are unable to seek refuge elsewhere; hard-substrate obligate i.e. those that live on polymetallic nodules will be the most at risk as the substrate they inhabit will be harvested, as was seen by Vanreusel *et al.*^12^.

Another factor that may regulate diversity and abundance in the eastern CCZ is food availability modulated by POC flux from the euphotic zone to the seafloor. Abyssal ecosystems are generally considered “food limited” because only a very small percentage of overlying export production reaches the abyss^9^. The structure of abyssal communities has been shown to be strongly influenced by POC flux; in fact a linear relationship is known to exist between POC flux and megafaunal abundance across abyssal environments^9^,^49^,^50^. The POC flux in the eastern CCZ is the highest in the entire region^44^,^51^ and has been postulated^9^,^12^, in combination with the large area of this region, to lead to relatively high local and regional species diversity. A similar relationship has been observed in terrestrial and other aquatic ecosystems^9^,^52^. However, POC flux in the eastern CCZ appears to be lower than that at the PAP (0.81–2.36 g C m^−2^ yr)^53^ or Station M (0.33–4.6 g C for 20 m^2^ 10 day mean)^54^ and this may result in a community dominated by megafauna small in size in the CCZ^9^. Although no direct biomass measurements were done in this study, it was clear that most of the megafaunal morphotypes, including corals and xenophyophores, were small (<10 cm). This contrasts with communities observed at the PAP and Station M where larger holothurians, which are rare in our CCZ images (less than 1% or 57 ind. ha^−1^), are the dominant megafaunal taxon^41^,^55^.

While the presence of polymetallic nodules and relatively high POC flux in the UK-1 contract area compared to the rest of the CCZ may be the dominant environmental drivers of megafaunal richness, abundance and diversity, other environmental factors may also play a role. Small topographical changes, e.g. hills and valleys, on the seafloor in this area, also likely play a role in structuring the megafaunal community as has been seen by Durden *et al.* (2014). Xenophyophores are an important part of the UK-1 benthos and earlier studies in the eastern Pacific and elsewhere have shown that they provide habitat structure that can be utilized by other organisms^56^,^57^. In the present study, ophiuroids were frequently observed in association with xenophyophores, as was noted previously on east Pacific seamounts^56^,^57^. Like their smaller foraminiferal relatives^58^, these large protists may also form an intermediate link in the trophic chain between bacteria and multicellular organisms and thereby make a significant contribution to carbon cycling^39^.

### Standardization of megafauna surveys using video and still imagery

The quality of ROV or AUV imagery varies with vehicle height above the seafloor, the resolution and light sensitivity of the camera system, stability and speed of the vehicle, and quality and orientation of lighting. Variations in image quality among megafaunal surveys are unavoidable given the differing availability of equipment and budgets across research projects. This complicates the comparison of megafaunal data across deep-sea studies, including past and ongoing work conducted in the CCZ, highlighting the need for detailed descriptions of equipment and methods to facilitate data standardization and statistically-rigorous regional comparisons. There are essentially two issues, (1) the recognition of what constitutes a “megafaunal” organism (e.g., for quantitative abundance counts), and (2) the assignment of individuals to morphotype “species,” for both quantitative ecology and assessment of species richness and ranges. Quality assurance in deep-sea video and image analyses has been discussed^19^,^59^,^60^ but there are not yet comprehensive published guidelines. Reference images and morphotype atlases should be made available as early as is feasible. In addition, authors must be explicit in their working definition of “megafauna,” including minimum dimensions of organisms surveyed and major taxa included. Durden *et al.*^61^ discuss recent criteria for recognising morphotypes when annotating seafloor data, but there are no agreed standards. Comparisons across studies are also complicated by the fact that separate species atlases are constructed by different research groups but rarely intercalibrated, while new objects are added to atlases on an ongoing basis. These problems are prevalent in CCZ analyses. Although the ISA co-sponsored a workshop for the standardization of megafauna identification from images that lead to the creation of an online atlas in 2014 (http://ccfzatlas.com/) and proved useful during this analysis, there is a dire need to update this online atlas with new imagery and ensure megafauna are properly identified with the help of taxonomists.

Because video and still imaging are the only approaches that can provide megafaunal survey data on the appropriate spatial scales (kilometers), it is critical that imaging surveys continue to assess the community structure, diversity and species ranges of megafauna across the CCZ. Greater emphasis on the standardization of survey approaches is needed; we present here our criteria as an iterative step towards regional-level standardization and synthesis of megafaunal community structure and biodiversity data. We will also publish our morphotype atlas to facilitate standardization of the putative morphotypes (Amon *et al.*, in prep.). Finally, it is critical to recognize that morphotype identifications from imagery ultimately must be ground-truthed with physical collection of megafaunal specimens to allow detailed morphological and DNA-sequence analyses. The limited collection of voucher specimens in the CCZ thus far has severely hampered the reliable estimation of species richness and species ranges. The lack of taxonomic sampling of the megabenthos continues to be an issue, but we highlight here that there is also still a need for standardization of the video survey methods themselves, which could form the basis of a useful advisory workshop for future studies across the CCZ.

## Study Site and Methods

**Figure 1:**
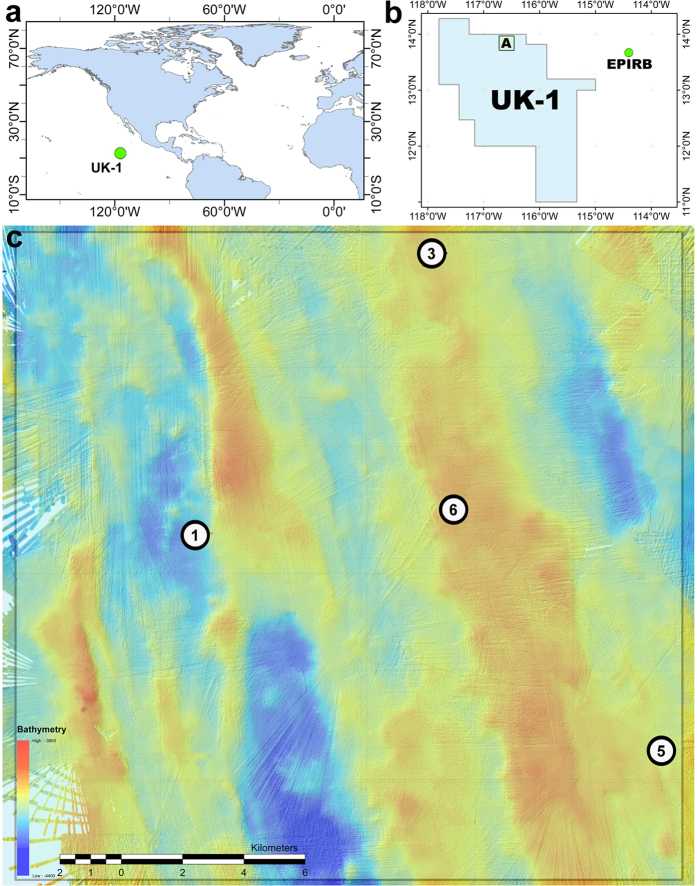
Locations of megafaunal surveys during ABYSSLINE cruise 01 (AB01) in the eastern Clarion-Clipperton Zone. (**a**) The location of the UK-1 exploration contract area in the Pacific Ocean. (**b**) The location of UK-1 Stratum A in relation to the UK-1 contract area and the AB01 ROV dive site, EPIRB, which was approximately 250 km east of the UK-1 contract area. (**c**) A bathymetric map of UK-1 Stratum A with the locations of ROV dives indicated by circles with the dive number. All maps were created by Seafloor Investigations Ltd. for the ABYSSLINE Project using ArcGIS software (https://www.arcgis.com/features/). (**c**) was created using unpublished ship-based bathymetry collected during AB01.

**Figure 2:**
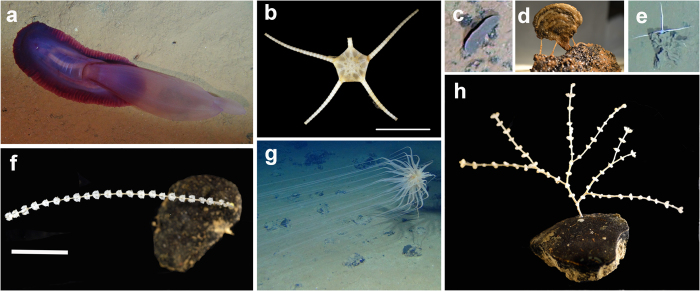
Dominant megafauna observed in the eastern CCZ. (**a**) *Psychropotes* cf. *semperiana*; (**b**) *Ophiomusium* cf. *glabrum*; (**c**) Xenophyophore plate-like morphotype 1: *Psammina* sp. *in situ* on seafloor; (**d**) Xenophyophore plate-like morphotype 1: *Psammina* sp. *in situ* close up; (**e**) Xenophyophore plate-like morphotype 8/9; (**f**) *Calyptrophora persephone*; (**g**) *Relicanthus* sp.; (**h**) *Abyssoprimnoa gemina.* Image attribution: (**a**,**c**,**e**,**g**) - DJ Amon & CR Smith, University of Hawai’i; (**d**) – AJ Gooday and A Goineau, National Oceanography Centre, Southampton; (**b**,**f**,**h**) – AG Glover, TD Dahlgren & H Wiklund, Natural History Museum, London & Uni Research.

**Figure 3:**
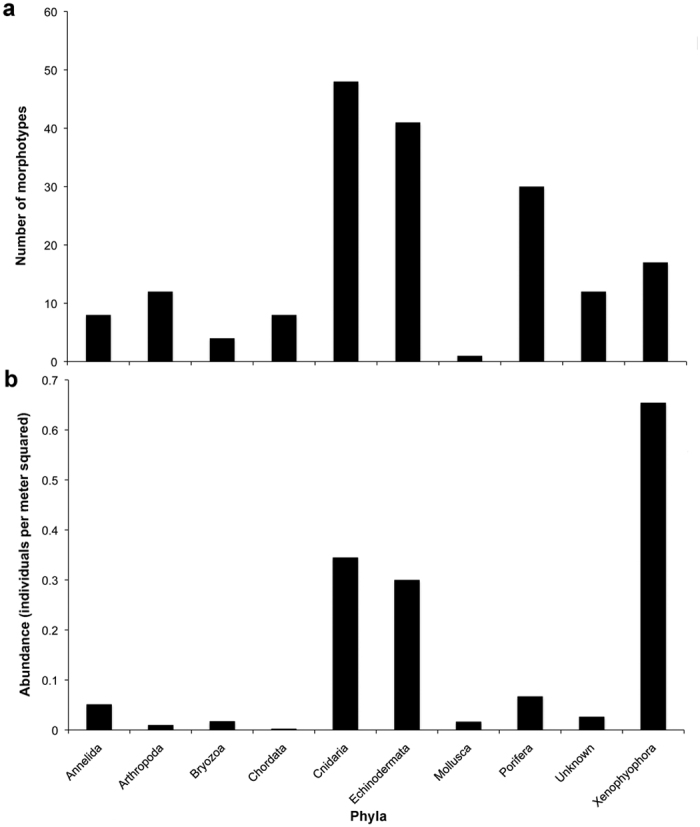
Megafaunal morphotype richness and individual abundance observed during AB01. (**a**) Megafaunal morphotype richness by phylum or other grouping in the case of xenophyophores. (**b**) Megafaunal abundance by phylum or other grouping in the case of xenophyophores. ‘Unknown’ refers to morphotypes that could not be assigned to a phylum.

**Figure 4:**
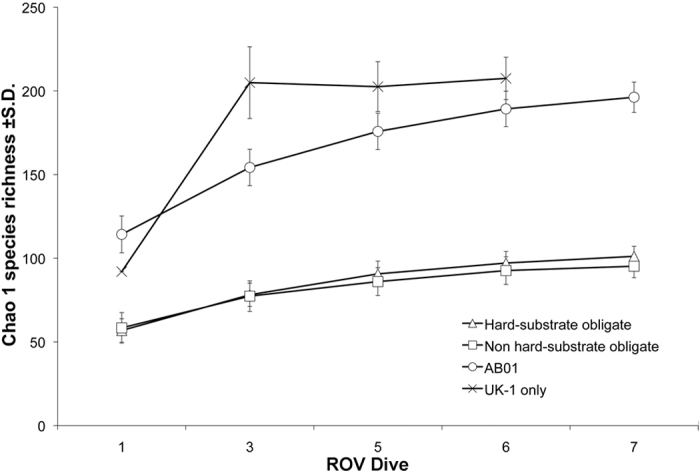
Chao-1 estimate of species richness (±s.d.) of epibenthic megafauna with increasing number of ROV dives during AB01 in the eastern CCZ. Circles = all megafauna surveyed during AB01 (five ROV dive sites); crosses = megafauna from only the UK-1 contract area (four ROV dive sites); triangles = megafauna observed only on hard substrata (nodules); squares = megafauna occurring on sediments or both sediments and nodules.

**Figure 5:**
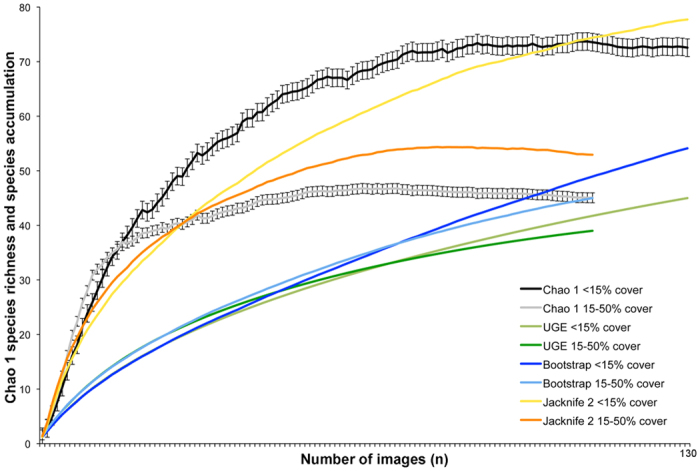
The relationship between species richness and nodule cover during AB01. Chao-1 species richness (±s.e.) in areas of low nodule percent cover (<15% cover) is represented by the black line, Chao 1 species richness (±s.e.) in areas of medium nodule percent cover (15–50% cover) is represented by the grey line. Ugland (UGE) species accumulation in areas of low nodule percent cover (<15% cover) is represented by the light green line and Ugland (UGE) species accumulation in areas of medium nodule percent cover (15–50% cover) is represented by the dark green line. Bootstrap species richness in areas of low nodule percent cover (<15% cover) is represented by the dark blue line, Bootstrap species richness in areas of medium nodule percent cover (15–50% cover) is represented by the light blue line. Jacknife 2 species richness in areas of low nodule percent cover (<15% cover) is represented by the yellow line, Jacknife 2 species richness in areas of medium nodule percent cover (15–50% cover) is represented by the orange line. High nodule percent cover (>50% cover) was not observed during this study.

The UK-1 exploration contract area is located in the eastern CCZ (Fig. 1) and has an annual POC flux to the abyssal seafloor of roughly 1 gCm^−2^ y^−1^, i.e., ~two-fold higher than in the western CCZ^50^. During the first cruise (ABYSSLINE Project cruise AB01, RV *Melville* cruise MV1313, 3–27 October, 2013), we focused on a 30 × 30 km stratum (UK-1 Stratum A) centered at 13°49′N, 116°36′W (Fig. 1). Multibeam bathymetric surveys during the cruise indicated an abyssal seafloor characterized by ridges and valleys running from NNW to SSE at 3900–4400 m depth (Fig. 1). Bottom-water temperatures were ~2 °C, bottom-water oxygen concentrations were ~3.2 ml L^−1^, and our observations revealed abyssal current velocities below sediment erosion thresholds throughout our 16 days on station (unpublished data, ABYSSLINE Project).

The commercial Remotely Operated Vehicle (ROV) *Remora III*, operated by Phoenix International Holdings, Inc., performed video surveys and sample collections at five randomly-located sites: four within UK-1 Stratum A, and one ~250 km to the east of the UK-1 contract area at a site here called ‘EPIRB’ centered at 13°40′N, 114°24′W (Fig. 1). Our original study design involved surveys at random sites within Stratum A, but ROV failures limited us to four sites. Work at the EPIRB site was dictated by an emergency response to an EPIRB distress signal and, although unplanned, provided a useful broader context for our study. The ROV was equipped with two manipulators, four ROS QLEDIII lights, one 1Cam Alpha Component high-definition downward-looking “science” video camera (1080p video and 24.1 megapixel stills) and one standard-definition forward-looking “pilot” video camera.

### Sample collection

Megafauna samples were collected by ROV manipulator, box corer, megacorer, and Brenke epibenthic sled, and were used for taxonomic identifications including ground-truthing identifications based on images. Once the respective sampling equipment was on deck, megafauna were quickly transferred to containers containing chilled seawater, photographed, and a tissue subsample taken for DNA analyses. In the case of metazoans, the DNA samples were preserved in 80% ethanol and the remainder of the animal was preserved in buffered 4% formalin-seawater solution or 95% ethanol, depending on the taxon. Xenophyophores (protists) were either preserved in 4% buffered formalin for morphological study, RNAlater^®^ for molecular analyses, or in a few cases, air-dried. After the cruise, morphological samples were sent to taxonomic experts for identification (see acknowledgements) and all metazoan specimens sequenced for a range of DNA markers at the Natural History Museum, London, with tissue samples subsequently archived and made openly-available for future taxonomic work^37^,^38^,^62^.

### ROV megafaunal video surveys

ROV still images and video were collected at all five sites for the “qualitative” analysis of megafauna (Fig. 1). This analysis utilized all video from both “pilot” and “science” cameras (covering roughly 8000 m^2^) whereas the quantitative analysis only used the high-definition imagery from the “science” camera. Due to ROV failures and altitude instability during surveys, we were able to obtain only four 1-km surveys, two at each of two sites (Sites 6 and EPIRB), of sufficient quality to be utilized in the quantitative assessment (Fig. 1 and see Supplementary Table S4). At each site, both transects were conducted along random headings, with the second transect beginning within 100 m of the end of the first.

### Image analyses

All video from both cameras on the ROV were viewed multiple times and frames archived of each identifiable megafaunal morphotype. The criteria used for selection of megafaunal morphotypes was that individuals were greater than 2 cm in maximum dimension and that there was sufficient detail to identify them to a putative ‘species-level’ morphotype (see Supplementary Figure S3). Morphotypes that could not be identified to species but appeared morphologically distinct were assigned a unique informal species name (e.g. Polynoidae sp. 1). Both metazoans and protists (xenophyophores) were included in this analysis. These were sorted into taxa, identified by taxonomic experts or by using the “Atlas of Abyssal Megafauna Morphotypes of the Clarion-Clipperton Fracture Zone” created for the ISA (http://ccfzatlas.com/), and then used to create an ABYSSLINE megafaunal morphotype atlas for the UK-1 contract area (Amon *et al.*, in prep). This UK-1 atlas was crucial for our quantitative faunal analyses and will also be useful for subsequent megafaunal analyses within the CCZ. This process provided an estimate of the number of megafaunal species in UK-1 Stratum A and the UK-1 contract area, and will aid in delimiting species ranges. However, since the majority of the morphotypes were not collected, it is impossible to confirm species identities in most cases or undertake systematic studies on this fauna. These issues are explored further in the discussion.

During surveys, the vehicle had substantial difficulty maintaining constant altitude, direction and velocity over the seabed, thereby limiting the availability of usable imagery. To facilitate quantitative analyses of abundance, the high-definition video from each of the four quantitative transects was split into frames taken every two seconds, using the software Quicktime Pro 7. This yielded approximately 13,700 frames, from which blurry images, images at altitudes >3.2 m and <1.2 m, and overlapping images were removed. The remaining 2458 frames were color-corrected, scaled, and analyzed in ImageJ^®^ (see Supplementary Table S4 and Supplementary Figure S3). In order to maximize the spatial coverage of our study, we enumerated and identified megafauna (using the criteria defined above) within the maximum usable area within each randomly-selected frame (quadrat sizes varied from 0.2 to 4.5 m^2^) (see Supplementary Table S4 and Supplementary Figure S3).

Because of the enormous effort required to count and measure the nodules in quantitative surveys (an important environmental variable in our study), exposed nodule abundance and size was evaluated in a subset (10%) of the frames used in the quantitative megafaunal analyses, referred to as quantitative nodule analysis from here forward. These frames were selected at random and then colour-corrected, scaled, and overlain with a randomly-placed quadrat of 0.66 m^2^ using ImageJ^®^ software. Within each seafloor quadrat, each nodule was counted and then manually outlined to measure plan area. From these values, percent nodule cover was calculated for each quadrat. All megafauna within the quadrat were counted, identified to morphotype using the criteria above, and their underlying substrate recorded. Although many of the nodules in the UK-1 contract area are partially or fully buried in sediment, our nodule counts could only record exposed nodule surfaces. These data reflect the quantity of sediment-free hard substrate available to megabenthos, e.g., for attachment sites.

### Statistical analyses

Species accumulation curves and richness estimates were made using Primer v.6^63^. Since Ugland species accumulation curves indicated that species inventories had not yet reached asymptotes and were continuing to accumulate, the recommendations of Magurran^64^ were followed and the Chao 1, Jacknife 2 and Bootstrap estimators were used to estimate total species richness.

Community similarities were evaluated using a cluster analysis based on Bray-Curtis similarities of square-root transformed abundance data (to allow contributions from both common and rare species) using Primer v.6 software. For these analyses, a large proportion of individuals including (for example) many ophiuroids, could not be confidently assigned to a species-level morphotype (e.g. *Ophiomusium* cf. *glabrum* and *Amphioplus daleus*). For similarity analyses, we assigned unidentified individuals to morphotypes in proportion to morphotype occurrence for confidently identified individuals from the same taxon. This method effectively split the unresolvable individuals into morphotypes in a proportion that was consistent with our confident observations. This approach likely underestimated the number of species present. The contributions to diversity of obligate hard-substrate-dwelling fauna, as well as fauna not requiring hard substrate (i.e., “facultative” species seen on both nodules and sediment, soft-sediment obligates, bentho-pelagic species), were assessed by determining the number of species unique to hard substrate (nodules), and by estimating total species richness by site using Chao-1 species-richness estimators.

For quantitative surveys, non-parametric Kruskal-Wallis comparisons were used to test for significant differences between megafaunal abundances and nodule parameters by image at site and transect levels. Correlations between faunal abundance and nodule parameters were also explored using Pearson’s product-moment correlation coefficient. Chi-squared tests were used to measure significant differences between faunal abundances, biodiversity indices (Shannon’s H’, Hulbert rarefaction Es(100) and Pielou’s Evenness J’) and nodule parameters at the site and transect level. A p-level = 0.05 was used throughout as the criterion for statistical significance. These analyses were conducted in SPSS Statistics v. 22.0 65.

## Additional Information

**How to cite this article**: Amon, D. J. *et al.* Insights into the abundance and diversity of abyssal megafauna in a polymetallic-nodule region in the eastern Clarion-Clipperton Zone. *Sci. Rep.* **6**, 30492; doi: 10.1038/srep30492 (2016).

## Supplementary Material

Supplementary Information

## Figures and Tables

**Table 1:** Diversity indices measured from AB01 surveys compared with other abyssal habitats.

	Abundance (s.e.) (m^−2^)	Number of morphotypes (S)	Pielou’s Evenness (J’)	Shannon’s Diversity (H’)	Hulbert Rarefaction (Es(100))
Site 6, Transect 1	1.28 (0.06)	88	0.73	3.26	31.94
Site 6, Transect 2	1.33 (0.05)	90	0.73	3.27	31.25
Site EPIRB, Transect 1	1.66 (0.06)	91	0.71	3.20	28.51
Site EPIRB, Transect 2	1.72 (0.07)	83	0.73	3.21	28.47
UK-1 Stratum A (Site 6)	1.31 (0.04)	110 (170)^&^	0.70	3.31	31.87
AB01 (Sites 6 and EPIRB)	1.48 (0.05)	136 (180)^	0.69	3.25	28.62
Station M	0.29–11.1	102	0.6–0.9	2.5–3.3	—
PAP	0.10–0.55	43	0.07–0.11	1.7–2.7	12.3–22.4

The AB01 values were obtained using only the imagery from the quantitative analyses. ^&^This value was obtained using all imagery from UK-1 Stratum A. ^This value was obtained using all imagery from AB01. *This value is for Es(103). Values for Station M are from Khunz *et al.* (2014) and PAP values are from Durden *et al.* (2015). s.e. = standard error.

**Table 2:** Dominant megafaunal species observed during AB01 in the eastern CCZ.

**Site 6, Transect 1**	Individuals m^−2^	% Abundance
*Ophiomusium* cf. *glabrum*	0.28	21.75
Xenophyophore plate-like morphotype 1: *Psammina* sp.	0.11	8.64
Xenophyophore plate-like morphotype 2	0.10	8.10
Xenophyophore plate-like morphotype 7	0.07	5.62
Xenophyophore plate-like morphotype 10	0.07	5.22
**Site 6, Transect 2**		
*Ophiomusium* cf. *glabrum*	0.23	17.15
Xenophyophore plate-like morphotype 2	0.16	11.73
Xenophyophore plate-like morphotype 1: *Psammina* sp.	0.11	8.68
Xenophyophore reticulated plate-like morphotype 6: *Reticulammina* sp.	0.08	6.31
Xenophyophore plate-like morphotype 8 or 9	0.08	6.10
**Site EPIRB, Transect 1**		
Primnoidae morphotype 2	0.18	10.92
*Abyssoprimnoa gemina*	0.18	10.86
*Ophiomusium* cf. *glabrum*	0.17	10.25
Xenophyophore plate-like morphotype 2	0.16	9.58
Xenophyophore plate-like morphotype 8 or 9	0.14	8.49
**Site EPIRB, Transect 2**		
Xenophyophore plate-like morphotype 2	0.21	12.34
*Abyssoprimnoa gemina*	0.21	12.28
*Ophiomusium* cf. *glabrum*	0.15	8.95
Xenophyophore plate-like morphotype 1: *Psammina* sp.	0.15	8.95
Xenophyophore plate-like morphotype: *Psammina limbata*	0.10	5.98
**Total Site 6**		
*Ophiomusium* cf. *glabrum*	0.26	19.47
Xenophyophore plate-like morphotype 2	0.13	9.90
Xenophyophore plate-like morphotype 1: *Psammina* sp.	0.11	8.66
Xenophyophore reticulated plate-like morphotype 6: *Reticulammina* sp.	0.07	5.66
Xenophyophore plate-like morphotype 7	0.07	5.19
**Total Site EPIRB**		
*Abyssoprimnoa gemina*	0.20	11.56
Xenophyophore plate-like morphotype 2	0.19	10.95
*Ophiomusium* cf. *glabrum*	0.16	9.60
Xenophyophore plate-like morphotype 1: *Psammina* sp.	0.14	8.22
Primnoidae morphotype 2	0.13	7.95
**Total for Study**		
*Ophiomusium* cf. *glabrum*	0.21	14.17
Xenophyophore plate-like morphotype 2	0.16	10.36
Xenophyophore plate-like morphotype 1: *Psammina* sp.	0.13	8.36
*Abyssoprimnoa gemina*	0.10	6.83
Xenophyophore plate-like morphotype 8 or 9	0.08	5.48

Species listed are the top five by percentage abundance.
