# Supplementary material for: Insights into the abundance and diversity of abyssal megafauna in a polymetallic-nodule region in the eastern Clarion-Clipperton Zone
Source: Sci Rep. 2016 Jul 29;6:30492. doi: 10.1038/srep30492 (PMC4965819; doi:10.1038/srep30492)
Supplement: Supplementary Information [file srep30492-s1.pdf]

Insights into the abundance and diversity of abyssal megafauna in a polymetallic-nodule region in the eastern Clarion-Clipperton Zone

Diva J. Amon[a*], Amanda F. Ziegler[a], Thomas G. Dahlgren[b,c], Adrian G. Glover[d], Aurélie Goineau[e], Andrew J. Gooday[e], Helena Wiklund[d], Craig R. Smith[a]

Supplementary Information

Supplementary Figure S1. Rank abundance curve for megafauna morphotypes observed during the AB01 quantitative surveys in the eastern CCZ.

Supplementary Figure S2. Similarity between the megafaunal communities observed on the AB01 quantitative transects. A cluster dendrogram using the group averages of results from Bray Curtis similarity tests performed on the species composition data from the four transects. Note that Site 6 and Site EPIRB are separated by 250 km.

Supplementary Figure S3. An example image from a transect undertaken at Site 6 in the UK-1 contract area. This image was quantitatively analysed during this study as can be seen by the quadrat and the fauna labelled with numbers (1= *Calyptrophora persephone*, 2= Xenophyophore plate-like morphotype 7).

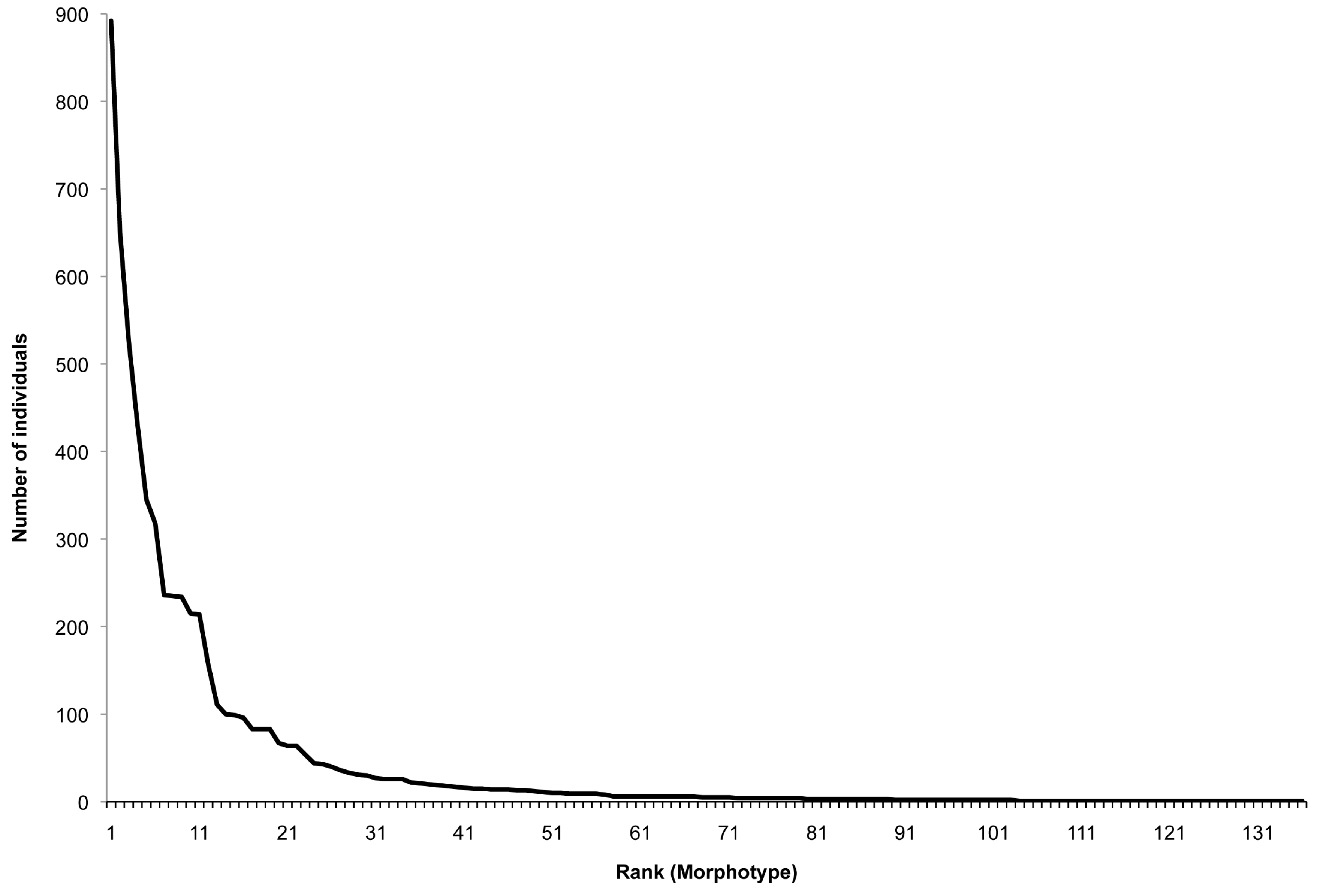

Number of individuals
900
800
700
600
500
400
300
200
100
0
1
11
21
31
41
51
61
71
81
91
101
111
121
131
Rank (Morphotype)

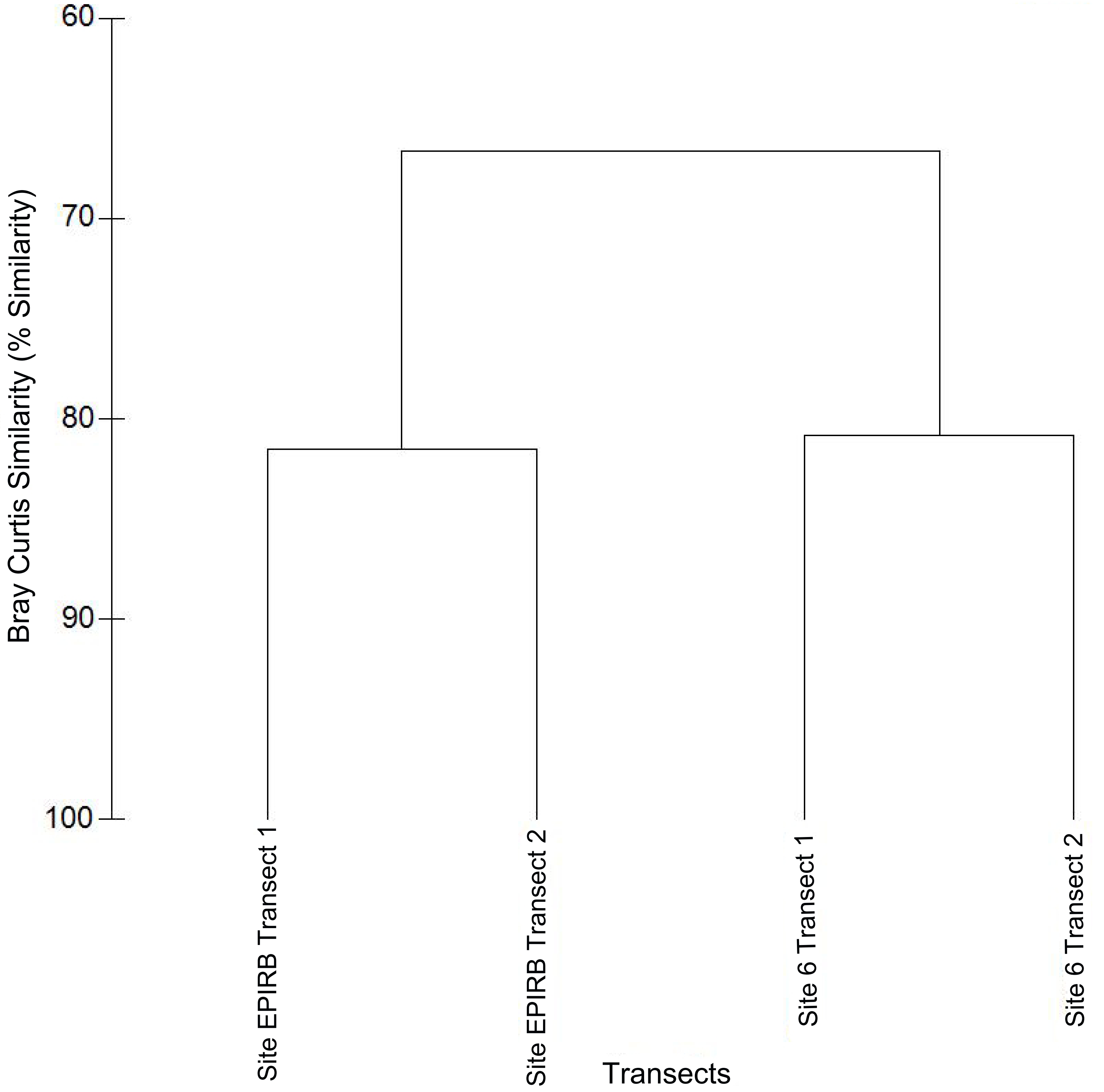

Bray Curtis Similarity (% Similarity)
60
70
80
90
100
Site EPIRB Transect 1
Site EPIRB Transect 2
Site 6 Transect 1
Site 6 Transect 2
Transects

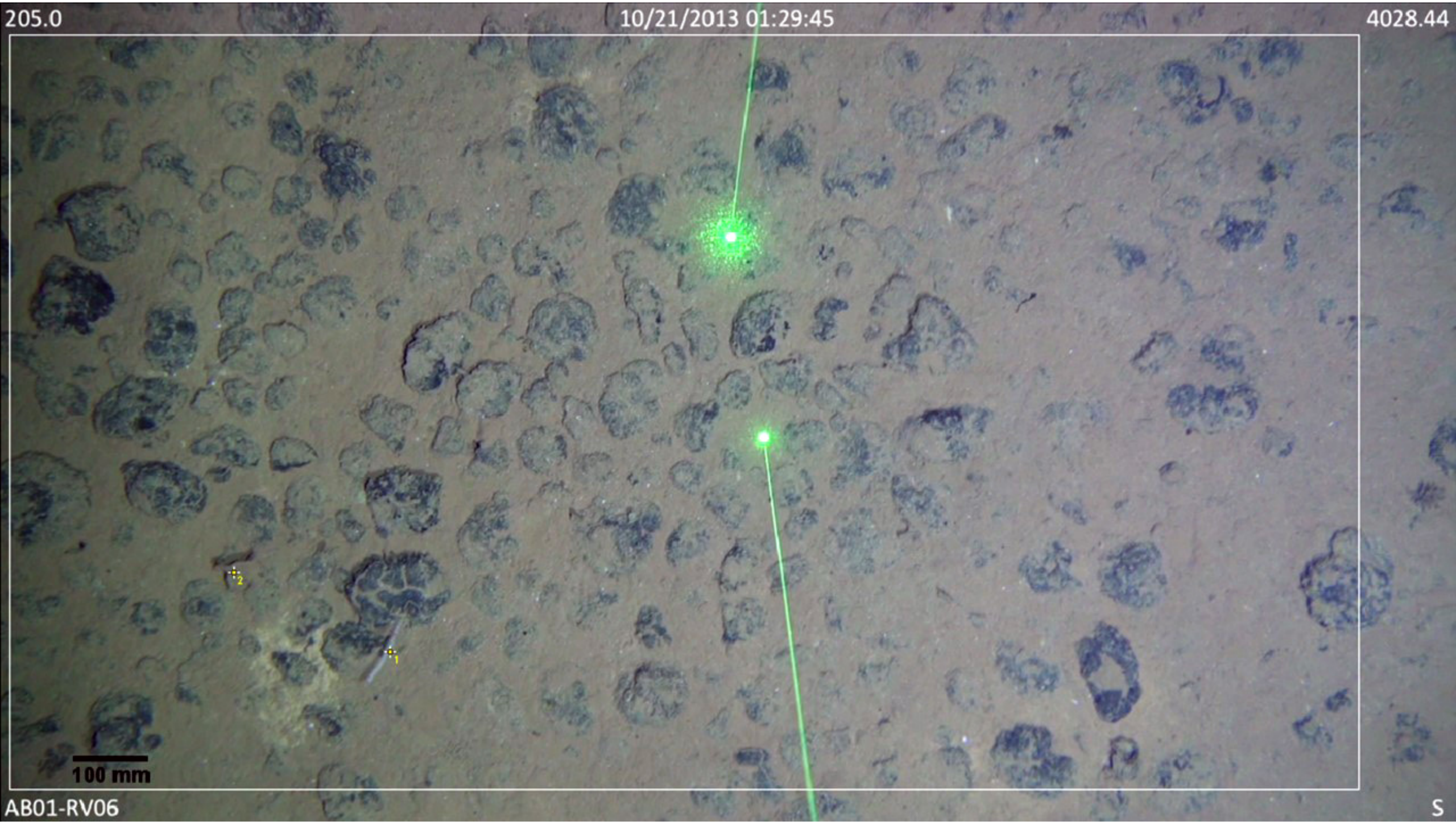
205.0
10/21/2013 01:29:45
4028.44
100 mm
AB01-RV06
S

Supplementary Table S1. Megafauna collected from the eastern CCZ during AB01. Information about the contract areas that each morphotype has previously been found in was taken from the Atlas of Abyssal Megafauna Morphotypes of the CCZ (**www.ccfzatlas.com**) and also from Chunsheng & Douding, 2002. These sources identified the fauna from images and thus may have inaccuracies. * indicates that this species was identified also collected from specimens from the BGR contract area. New species are in bold.

**Phylum**	**Class**	**Subclass/ Order**	**Family**	**Genus and Species/Morphotype**	**Authority**	**Latitude**	**Longitude**	**Depth Sampled During AB01**	**Known Locations Outside of Abyssal CCZ**	**Known Exploration Contract Area**
Cnidaria	Anthozoa	Hexacorallia	Hormathiidae	***Phelliactis* n. sp. 1**	-	13°51.872	116°32.893	4025m	-	UKSRL
		Octocorallia	Isididae	**(Mopseinae) n. gen. 1**	-	13°45.623	116°28.076	4043m	-	UKSRL
				**n. gen. 2**	-	13°51.874	116°32.894	4026m	North Pacific Ocean	UKSRL, BGR, IFREMER, NORI
			Primnoidae	***Abyssoprimnoa gemina***	Cairns, 2015	13°45.700	116°27.620	4111m	-	UKSRL, BGR*, NORI
				Calyptrophora persephone	Cairns, 2015	13°43.597	116°40.200	4160m	-	UKSRL, BGR*, IFREMER
Echinodermata	Asteroidea	Brisingidae	Freyellidae	*Freyastera* cf. *benthophila*	Sladen, 1889	13°51.734	116°32.773	4011m	Pacific, Atlantic, Indian Oceans	UKSRL, BGR, COMRA, DORD, IFREMER, IOM, YMG
	Holothuroidea	Elasipodida	Psychropotidae	*Benthodytes sanguinolenta*	Théel, 1882	13°57.774	116°33.078	4063m	Pacific, Atlantic, Southern Oceans	UKSRL
				Psychropotes semperiana	Théel, 1882	13°57.768	116°33.056	4062m	Pacific, Atlantic Oceans	UKSRL, BGR, COMRA, IFREMER, IOM
	Ophiuroidea	Ophiurida	Ophiolepididae	*Ophiomusium* cf. *glabrum*	Lütken & Mortensen, 1899	13°45.500	116°41.911	4081m	East Pacific Ocean	UKSRL, BGR, COMRA, DORD, IFREMER, IOM, NORI, YMG
				Ophiomusium cf. *glabrum*	same as above	13°57.774	116°33.078	4063m	same as above	same as above
				Ophiomusium cf. *glabrum*	same as above	13°45.621	116°28.074	4043m	same as above	same as above
				Ophiomusium cf. *glabrum*	same as above	13°45.707	116°27.601	4111m	same as above	same as above
				Ophiomusium cf. *glabrum*	same as above	13°51.872	116°32.893	4025m	same as above	same as above
				Ophiomusium cf. *glabrum*	same as above	13°51.893	116°32.907	4026m	same as above	same as above
				Ophiomusium cf. *glabrum*	same as above	13°40.768	114°24.873	3952m	same as above	same as above
			Amphiuridae	*Amphioplus (Unioplus) daleus*	Lyman, 1879	13°51.801	116°32.799	4053m	Pacific, Atlantic Oceans	UKSRL
Porifera	Hexactinellida	Lyssacinosida	Rossellidae	***Caulophacus* n. sp. 1**	-	13°50.997	116°38.746	3952m	-	-
				***Caulophacus* n. sp. 2**	-	13°40.786	114°24.873	4110m	-	UKSRL

Supplementary Table S2. Megafaunal morphotypes observed from all imagery collected during AB01 in the eastern CCZ. Presence within the UK-1 contract area and at the EPIRB site approximately 250 km to the east of the UK-1 contract area is indicated.

Phylum	Class	Order	Family	Genus	Morphotype	UK-1 Contract Area	EPIRB Site
Annelida	Polychaeta				sp. 1	X	-
		Phyllodocida	Polynoidae		sp. 1	X	X
		Sabellida	Sabellidae		sp. 1	X	X
					sp. 2	X	X
					sp. 3	X	X
					sp. 4	X	X
			Serpulidae		sp. 1	X	X
					sp. 2	X	X
Arthropoda	Malacostraca	Decapoda		(juvenile?)	sp. 1	X	X
					sp. 2	X	-
					sp. 3	X	X
			Aristeidae	*Plesiopenaeus*	*armatus*	X	X
			Nematocarcinidae	*Nematocarcinus*	sp. 1	X	X
			Solenoceridae	*Hymenopenaeus*	*nereus*	X	X
		Amphipoda			sp. 1	X	-
					sp. 2	X	X
					sp. 3	X	-
		Isopoda	Eurycopinae		sp. 1	X	X
	Maxillopoda	Scalpelliformes			sp. 1	X	X
	Pycnogonida				sp. 1	X	-
Bryozoa					sp. 1	X	X
					sp. 2	X	-
	Stenolaemata	Cyclostomatida			sp. 1	X	X
					sp. 2	X	X
Chordata	Ascidiacea				sp. 1	X	-
		Phlebobranchia	Octacnemidae	*Megalodicopia*	sp. 1	-	X
	Actinopteri	Aulopiformes	Bathysauridae	*Bathysaurus*	cf. *mollis*	X	-
			Ipnopidae	*Ipnops*	cf. *meadi*	X	X
		Gadiformes	Macrouridae	*Coryphaenoides*	*armatus/yaquinae*	X	X
		Ophidiiformes	Ophidiidae		sp. 1	X	X
				Bassozetus	cf. *nasus*	X	X
		Perciformes	Zoarcidae	*Pachycara*	cf. *bulbiceps*	X	X
Cnidaria	Anthozoa	Actiniaria			sp. A	X	-
					sp. B	X	-
					sp. C	X	-
					sp. F	X	-
					sp. G	X	-
					sp. H	-	X
					sp. I	X	X
					sp. J	-	X
					sp. K	-	X
					sp. L	X	X
					sp. M	X	-
					sp. N	X	X
					sp. O	-	X
					sp. 2	X	X
					sp. 4	X	X
					sp. 8	X	X
					sp. 10	X	-
					sp. 12	X	-
					sp. 14	X	X
					sp. 15	X	X
			Hormathiidae	*Phelliactis*	sp. E	X	-

		Antipatharia	Schizopathidae		sp. 1	X	X
				Abyssopathes	cf. *lyra*	X	X
				Bathypathes	sp. 1	X	X
					sp. 2	X	-
					cf. *alternata*	X	-
					cf. *patula*	X	X
		Ceriantharia			sp. 1	X	X
		Corallimorpharia	Corallimorphidae	*Corallimorphus*	sp. 1	X	X
		Incerti ordinis	Relicanthidae	*Relicanthus*	sp. 1	-	X
		Zoantharia	Zoanthidae		sp. 1	X	X
		Alcyonacea			sp. 1	X	X
			Isididae		sp. 1	X	X
					sp. 2	-	X
				(Mopseinae)	sp. 1	X	X
				(Mopseinae)	sp. 2	X	X
				n. gen	n. sp.	X	X
			Primnoidae	*Calyptrophora*	*persephone*	X	X
				Abyssoprimnoa	*gemina*	X	X
					sp. 2	X	X
		Pennatulacea			sp. 1	-	X
			Umbellulidae	*Umbellula*	sp. 1	X	X
	Scyphozoa				sp. 1	X	-
					sp. 2	X	-
		Coronatae	Periphyllidae	*Periphylla*	cf. *periphylla*	X	-
		Trachymedusae	Halicreatidae		sp. 1	X	-
			Rhopalonematidae		sp. 1	X	X
					sp. 2	-	X
Echinodermata	Asteroidea				sp. 1	X	X
		Velatida	Pterasteridae		sp. 1	X	X
					sp. 2	X	X
				Hymenaster	sp. 1	X	X
					sp. 2	X	-
		Paxillosida			sp. 1	X	-
			Porcellanasteridae		sp. 1	X	X
				Porcellanaster	sp. 2	X	X
		Brisingida	Freyellidae	*Freyastera*	cf. *benthophila*	X	X
				Freyella	sp. 1	X	X
	Crinoidea				sp. 1	X	X
		Bourgueticrinida			sp. 1	X	X
					sp. 2	X	X
		Hyocrinida	Hyocrinidae		sp. 1	X	X
		Comatulida			sp. 1	X	X
	Echinoidea	Aspidodiadematoida	Aspidodiadematidae	*Plesiodiadema*	sp. 1	X	-
		Holasteroida			sp. 1	X	-
			Pourtalesiidae	*Cystocrepis*	sp. 1	X	X
				Cytocrepis/Echinocrepis	sp. 1	X	-
			Urechinidae	*Cystechinus/Urechinus*	sp. 1	X	X
	Holothuroidea	Aspidochirotida	Mesothuriidae	*Mesothuria*	sp. 1	X	X
			Synallactidae	*Synallactes*	sp. 1	X	X
					sp. 2	X	X
					sp. 3	X	-
				(juvenile)	sp. 1	X	-
				Pseudostichopus	sp. 1	X	X
		Elasipodida	Deimatidae	*Oneirophanta*	sp. 1	X	-
			Elpidiidae	(juvenile)	sp. 1	X	-
				Amperima	sp. 1	X	X
				Peniagone	cf. *leander*	X	X
			Laetmogonidae		sp. 1	X	-
				Psychronaetes	cf. *hanseni*	X	-

			Psychropotidae	(juvenile)	sp. 1	X	X
				Benthodytes	cf. *incerta*	X	X
				Benthodytes	*sanguinolenta/typica*	X	X
				Psychropotes	*semperiana*	X	-
					cf. *verrucosa*	X	-
	Ophiuroidea	Ophiurida	Ophiuridae	*Amphiophiura*	cf. *bullata*	X	X
			Amphiuridae	*Amphioplus (Unioplus)*	*daleus*	X	X
			Ophiacanthidae	*Ophiacantha/Astrodia*	sp. 1	X	X
			Ophiolepididae	*Ophiomusium*	cf. *glabrum*	X	X
Mollusca	Bivalvia				sp. 1	X	X
Porifera					sp. 1	X	-
					sp. 2	X	-
					sp. 3	X	X
					sp. 4	X	X
					sp. 5	X	-
					sp. 6	X	-
					sp. 7	X	X
					sp. 8	X	X
					sp. 9	X	X
					sp. 10	X	-
					sp. 11	X	X
					sp. 12	X	X
					sp. 13	X	X
					sp. 14	-	X
					sp. 15	X	X
	Demospongiae	Astrophorida	Geodiidae	*Geodia*	sp. 1	X	X
	Hexactinellida	Amphidiscosida	Hyalonematidae	*Hyalonema*	sp. 1	X	X
					sp. 2	X	X
					sp. 4	X	X
		Hexactinosida	Euretidae	*Chonelasma*	cf. *choanoides*	X	X
			Farreidae	*Farrea*	sp. 1	X	X
		Lyssacinosida	Rosellidae	*Caulophacus*	sp. 1	X	X
					sp. 2	X	-
					sp. 4	X	-
				Rhabdocalyptus	sp. 1	X	X
			Euplectellidae		sp. 1	X	X
					sp. 2	X	X
				Docosaccus	cf. *maculatus*	X	X
				Euplectella	sp. 1	X	-
					cf. *suberea*	X	X
Unknown					sp. 1	X	X
					sp. 3	X	X
					sp. 4	X	X
					sp. 5	X	-
					sp. 6	X	-
					sp. 7	X	-
					sp. 9	X	X
					sp. 10	X	X
					sp. 11	X	-
					sp. 12	X	X
					sp. 14	X	X
Foraminifera	Xenophyophora				tubular sp. 2	X	X
					plate like sp. 1 - *Psammina* sp.	X	X
					cf. *Psammina limbata*	X	X
					plate like sp. 2	X	X
					plate like sp. 4	X	X
					plate like sp. 5	X	X
					reticulated plate like	X	X

sp. 6 - Reticulammina		
plate like sp. 7	X	X
plate like sp. 8 or 9	X	X
plate like sp. 10	X	X
plate like sp. new	X	X
reticulate sp. 1	X	X
reticulate sp. 2	X	X
reticulate sp. 4	X	X
reticulate sp. 5	X	X
reticulate sp. 6	X	X
reticulate sp. 8	X	X

Supplementary Table S3. Nodule parameters by AB01 transect and site in the eastern CCZ. The mean exposed nodule plan area was calculated by finding the mean nodule plan area in each image and then a further mean taken at the transect or dive level. ±s.e. = standard error and the ranges are displayed in brackets.

	**Mean Exposed Nodule Plan Area / cm^2 ±s.e. (range)**	**Mean Nodule Abundance / Nodules m^{-2} ±s.e. (range)**	**Mean % Cover of Nodules / ±s.e. (range)**	**Mean Megafaunal Abundance / Fauna m^{-2} ±s.e.**
**Site 6, Transect 1**	17.28 ±2.22 (0.68-197.59)	84.49 ±6.73 (22.73-306.06)	14.60 ±0.73 (5.05-30.82)	1.85 ±0.21
**Site 6, Transect 2**	10.78 ±1.55 (0.48-691.23)	133.38 ±13.26 (3.03-428.79)	14.38 ±1.32 (0.21-42.45)	1.67 ±0.22
**Site EPIRB, Transect 1**	10.85 ±3.12 (0.31-1041.44)	190.05 ±22.02 (3.00-534.85)	20.61 ±1.73 (0.54-49.99)	2.00 ±0.25
**Site EPIRB, Transect 2**	8.31 ±1.46 (0.29-193.29)	197.79 ±21.22 (21.21-516.67)	16.92 ±1.36 (2.84-48.79)	3.25 ±0.37
**Site 6, Transects pooled**	13.14 ±1.31 (0.48-691.23)	110.22 ±7.89 (3.03-428.79)	14.48 ±0.77 (0.21-42.45)	1.75 ±0.15
**Site EPIRB, Transects pooled**	9.68 ±1.84 (0.29-1041.44)	193.49 ±15.38 (3.00-516.67)	19.18 ±1.15 (0.54-49.99)	2.55 ±0.22

Supplementary Table S4. ROV transect locations during AB01. Site 6 was within UK-1 Stratum A, whereas Site EPIRB was located approximately 250 km to the east of the UK-1 contract area.

**ROV Site**	**Transect**	**Start Latitude**	**Start Longitude**	**Start Depth (m)**	**End Latitude**	**End Longitude**	**End Depth (m)**	**Number of Quantitative Images**	**Seafloor Area Surveyed (m^2)**
6	1	13°51.813'N	116°32.779'W	4028	13°51.294'N	116°32.863'W	4021	641	1163
	2	13°51.318'N	116°32.878'W	4029	13°51.839'N	116°32.919'W	4026	715	1105
EPIRB	1	13°40.766'N	114°24.881'W	3951	13°40.705'N	114°24.347'W	3906	603	993
	2	13°40.755'N	114°24.337'W	3906	13°40.763'N	114°24.884'W	3950	499	941
